# A genetic correlation and bivariate genome-wide association study of grip strength and depression

**DOI:** 10.1371/journal.pone.0278392

**Published:** 2022-12-15

**Authors:** Tianhao Zhang, Lujun Ji, Jia Luo, Weijing Wang, Xiaocao Tian, Haiping Duan, Chunsheng Xu, Dongfeng Zhang

**Affiliations:** 1 Department of Epidemiology and Health Statistics, Public Health College, Qingdao University, Qingdao, Shandong Province, China; 2 Qingdao Municipal Center for Disease Control and Prevention, Qingdao Institute of Preventive Medicine, Qingdao, Shandong, China; Peking University, Institute of Mental Health, CHINA

## Abstract

Grip strength is an important biomarker reflecting muscle strength, and depression is a psychiatric disorder all over the world. Several studies found a significant inverse association between grip strength and depression, and there is also evidence for common physiological mechanisms between them. We used twin data from Qingdao, China to calculate genetic correlations, and we performed a bivariate GWAS to explore potential SNPs, genes, and pathways in common between grip strength and depression. 139 pairs of Dizygotic twins were used for bivariate GWAS. VEAGSE2 and PASCAL software were used for gene-based analysis and pathway enrichment analysis, respectively. And the resulting SNPs were subjected to eQTL analysis and pleiotropy analysis. The genetic correlation coefficient between grip strength and depression was -0.41 (-0.96, -0.15). In SNP-based analysis, 7 SNPs exceeded the genome-wide significance level (*P*<5×10^−8^) and a total of 336 SNPs reached the level of suggestive significance (*P*<1×10^−5^). Gene-based analysis and pathway-based analysis identified genes and pathways related to muscle strength and the nervous system. The results of eQTL analysis were mainly enriched in tissues such as the brain, thyroid, and skeletal muscle. Pleiotropy analysis shows that 9 of the 15 top SNPs were associated with both grip strength and depression. In conclusion, this bivariate GWAS identified potentially common pleiotropic SNPs, genes, and pathways in grip strength and depression.

## Introduction

Grip strength, which can reflect muscle strength to some extent [1] and is correlated with nutritional status [2], disease status, and all-cause mortality [3], is a very important biomarker of aging [4]. Depression is a common psychiatric disorder that can significantly decrease the quality of life of older adults [5], leading to a heavy burden of disease worldwide [6]. Several previous cross-sectional [7, 8] and cohort [9, 10] studies have found a significant inverse association between grip strength and depression, and there is also evidence for common physiological mechanisms between grip strength and depression. For example, the muscle can release inflammatory cytokines such as IL-6, IL-8, and IL-15 [11]. Some studies have shown that weak skeletal muscle strength is related to an increase in serum proinflammatory cytokines [12, 13], which can lead to depression [14]. Furthermore, lower grip strength and depression have been shown to be associated with shortened cellular telomere length [15, 16]. In addition, physical activity can have a positive effect on serotonin [17], dopamine [18], and norepinephrine levels [19], which are closely related to depression.

In addition to environmental factors, genetic factors also play a very important role in the relationship between grip strength and depression. An elderly twin study found a heritability of 35% for hand-grip strength [20]. In the twin study by Tian et al., handgrip strength was found to have a moderate heritability of 59.68% [21]. The results of one meta-analysis showed a heritability of 56% (95% CI: 0.46–0.67) for isometric grip strength [22]. A large twin study in Sri Lanka found that the heritability of broadly defined depression was 61% in women [23]. Through a meta-analysis of twin studies, the heritability of depression was found to range from 31% to 48% [24]. However, there are no studies on the genetic correlation of grip strength with depression.

To date, little is known about shared genetic variation in depression and grip strength. Some overlapping results were found between depression and grip strength by contrasting univariate studies. Two cohort studies in older Swedish adults found that the APOEε4 allele was simultaneously associated with decreased grip strength and depression [25, 26]. In addition, serval studies have shown that the vitamin D receptor (*VDR*) gene is associated with muscle strength and senile depression symptoms [27–29]. The results of these univariate studies indirectly support the existence of common susceptibility genes for grip strength and depression, but there is still a lack of direct evidence. Compared with univariate research, a bivariate genome-wide association study (GWAS) can add two variables to the model simultaneously to find the potential pleiotropic genetic variation between phenotypes. Bivariate GWASs have higher statistical ability and more accurate parameter estimation than univariate studies [30, 31]. In addition, twin samples are more effective for studies targeting complex phenotypes than general population samples [32].

In summary, we hypothesized that grip strength and depression are regulated by common genetic factors. Therefore, we used twin data from Qingdao, China to calculate genetic correlations, and we performed a bivariate GWAS to explore potential single nucleotide polymorphisms (SNPs), genes, and pathways in common between grip strength and depression.

## Methods

### Study population

We used a twin sample from the Qingdao Twin Registration System in China, and the details of the sample can be found in previous literature [33]. Blood was collected from participants in a fasted state, and zygosity was determined by sex, blood type, and microsatellite DNA gene scanning with typing techniques. Participants differing in sex or blood type were identified as dizygotic (DZ) twins. Monozygotic (MZ) twins were identified when both sex and blood type was the same and all 15 short tandem repeats (STRs) were concordant. Twins fulfilling the following criteria could be included in the study: 1) older than 18 years; 2) available for follow-up; and 3) had blood samples, questionnaires, and phenotypic measurement data. We excluded twins if they 1) were pregnant or lactating; 2) were missing key indicator information; 3) had a critical illness or were unable to complete the survey; or 4) were professional athletes. Ultimately, 235 MZ and 134 DZ twins were included in the study.

### Phenotypes

Information such as sex and age were collected by questionnaires. The 30-item Geriatric Depression Scale (GDS-30, Chinese version) was used to assess depressive symptoms. The scale consists of 30 questions with a total score ranging from 0–30, with higher scores representing more severe depressive symptoms. This scale has been adapted precisely for the assessment of depression in middle-aged and older adults, and it is appropriate for the Chinese population [34]. Physical examinations were performed by trained investigators. Participants were required to squeeze the grip of the handgrip dynamometer (WCS-100, Nantong, China) as strongly as they could, with each hand tested 3 times. Like some other studies [35, 36], the maximum value was taken as the grip strength for analysis.

### Genetic correlations

We used Mx software to construct a bivariate Cholesky decomposition model. The classical twin model decomposes the total phenotypic variation into additive genetic effect (A), common environment effect (C), and special environment effect (E). The likelihood ratio chi-square test was used to compare the difference between the full model and its nested model. If a *P* value greater than 0.05 indicated that the full model was not significantly different from its nested model, the nested model was selected according to the Akaike information criterion (AIC) and the minimalist principle.

### Genotyping, quality control, and imputation

We used Infinium Omni2.5Exome-8v1.2 BeadChip from Illumina for genotyping DZ twins. The chip covers a wide range and has a good typing detection rate. After typing detection, SNPs were first quality-controlled, and individual SNPs should meet: 1) Locus missing rate < 0.05 (SNPs with high missing rates were difficult to genotype); 2) Calling rate > 0.98 (The low calling rate reflects the poor quality of the sample); 3) Hardy-Weinberg equilibrium (HWE) > 1×10^−6^ (HWE is a tool for tagging SNPs with a large number of genotyping errors); 4) Minor allele frequency (MAF) >0.05 (Variants with very low MAF are more susceptible to genotyping errors, as the rarer alleles occur in only a few individuals.). Then, the quality-controlled SNPs were imputed with the use of IMPUTE2 software [37], based on linkage disequilibrium (LD) principles with data from the third phase of the 1000 Genomes Project (ASIAN) [38] as the reference. The SNPs were quality controlled again after imputation with the standards of 1) a Hardy-Weinberg equilibrium (HWE)>1×10^−6^; 2) a minor allele frequency (MAF)>0.05; 3) information contents (info> = 0.9). Finally, 7,165,663 SNPs were used in the bivariate GWAS.

### Bivariate GWAS

#### SNP-based analysis

To explore the association of SNPs in handgrip-depression pairs, we used genome-wide efficient mixed-model association (GEMMA) [39], adjusting for sex, age, and BMI. Rank transformation based on Blom’s formula was used to normalize the skewed distributions of handgrip strength and depression. GEMMA fitted a multivariate linear mixed model (mvLMM) while controlling for relatedness and population structure to test marker associations of handgrip strength with depression. The significance level was defined as a *P* value<5×10^−8^ as a conventional Bonferroni-corrected threshold [40]. The suggested level of association was a *P* value<1×10^−5^, a commonly utilized threshold in GWASs [41]. The quantile-quantile (Q-Q) plot was used to visualize the population stratification, and the Manhattan plot was used to visualize the *P* value for each SNP on each chromosome.

#### Gene-based analysis

Versatile Gene-based Association Study-2 (VEGAS2) software [42] was used for gene-based analysis with the “1000G East ASIAN Population” as a reference. A total of 21,221 genes were tested so that the Bonferroni-corrected significance threshold was a *P* value<2.36×10^−6^(0.05/21,221). The nominal significance level was a *P* value<0.05 [43].

#### Pathway enrichment analysis

Pathway Scoring Algorithm (PASCAL) software [44] was used to evaluate pathway scores for pathway enrichment analysis. First, SNP loci were located in genes, and the association scores of all genes in a pathway were calculated. The chi-square and empirical scores were used to evaluate high-scoring pathways. Then, we obtained access information from the KEGG, BioCarta, and Reactome databases.

### Expression quantitative trait locus (eQTL) analysis

For the top 60 SNPs that reached the suggested level of significance, we examined their functionality using data from the GTEx portal (version 8) [45]. A *P* value<0.05 was considered significant in the single-tissue eQTL analysis. The posterior probability m-value that the eQTL effect existed in each tissue of a cross-tissue meta-analysis higher than 0.9 indicated that the tissue having had an eQTL effect [46].

### Pleiotropy analysis

We performed genetic pleiotropy tests on the top 15 SNPs that were most significant before imputation using the R package “pleio”, thus verifying whether they were indeed associated with both grip strength and depression.

### Ethics statement

This study conformed to the declaration of Helsinki, and all participants provided written informed consent. This study was approved by the Regional Ethics Committee of the Qingdao Center for Disease Control. And the decision reference number was 2012–01.

## Results

### Basic characteristics

The basic characteristics of the twin are shown in Table 1. A total of 369 pairs of twins were enrolled in this study. Among them, 134 were DZ twins and 235 were MZ twins. There were 362 males and 376 females. In the total sample, the median (interquartile range) age was 50 (45, 57), and the median (interquartile range) grip strength and depression scores of participants were 30.4 (23.8, 40.2) and 7 (4, 11), respectively. In the DZ twin sample, the median (interquartile range) age was 49 (45, 56), and the median (interquartile range) grip strength and depression scores of the participants were 31.1 (25.2, 42.5) and 7 (4, 11), respectively.

**Table 1:** Characteristics of participants by sex.

	Variables	Male		Female		Total population	
		N	M (Q)	N	M (Q)	N	M (Q)
Total sample	Age (year)	362	50 (45, 58)	376	50 (46, 56)	738	50 (45, 57)
	Grip strength	362	40.8 (34.8, 47.7)	376	24.4 (21.3, 27.7)	738	30.4 (23.8, 40.2)
	Depression score	362	7 (3.75, 11)	376	7 (4, 10)	738	7 (4, 11)
DZ twins	Age (year)	138	49 (45, 57)	130	49 (45, 56)	268	49 (45, 56)
	Grip strength	138	42 (36.9, 49.9)	130	25.2 (22.2, 28.6)	268	31.1 (25.2, 42.5)
	Depression score	138	7 (3, 11)	130	6 (4, 10)	268	7 (4, 11)

M: median; Q: quartile.

### Genetic correlations

As shown in Table 2, the phenotypic correlation coefficient between grip strength and depression was -0.27 (*P*<0.001), and the best fitting Cholesky decomposition model (ACE) identified that the genetic correlation coefficient between the two phenotypes was -0.41 (-0.96, -0.15). This finding suggested a moderate genetic correlation between grip strength and depression. The common and special environmental correlation coefficients were insignificant.

**Table 2:** Genetic correlations between grip strength and depression.

Phenotypic	Model	r_G_ (95%CI)	r_C_ (95%CI)	r_E_ (95%CI)	Phenotypic correlation coefficient	-2LL	Δdf	χ^2^	*P*
Grip strength–depression	ACE	-0.41 (-0.96, -0.15)	-1.00 (-1.00, 1.00)	-0.04 (-0.17, 0.09)	-0.27 (-0.34, -0.19)	3610.99			
	AE	-1.00 (-1.00, -0.78)	-	-0.12 (-0.23, -0.01)	-0.24 (-0.31, -0.16)	3618.14	1	7.153	0.007

r_G_: genetic correlation coefficient; r_C_: common environmental correlation coefficient; r_E_: special environmental correlation coefficient; -2LL: double negative logarithmic likehood function value; df: free degree.

### Bivariate GWAS

#### SNP-based analysis

**Fig 1:**
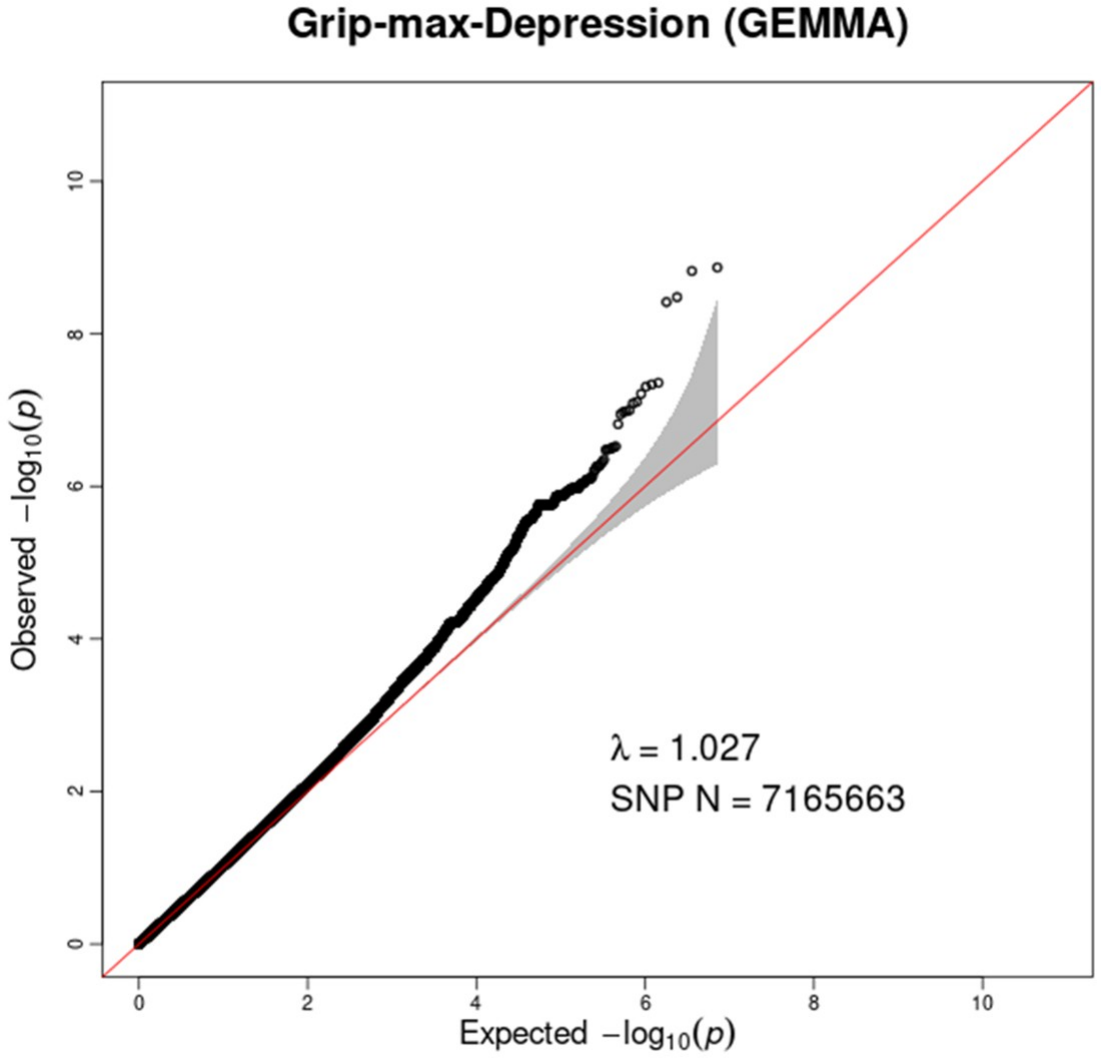
Quantile-quantile plot for bivariate genome-wide association study of grip strength and depression. The horizontal axis represents the expected -log_10_ (*P*), while the vertical axis represents the observed -log_10_ (*P*). The red line represents the expectation of the null hypothesis of no association, and the gray shaded area represents 95% confidence intervals of the null hypothesis. The black dots represent the observed data, and λ indicates genomic inflation.

**Fig 2:**
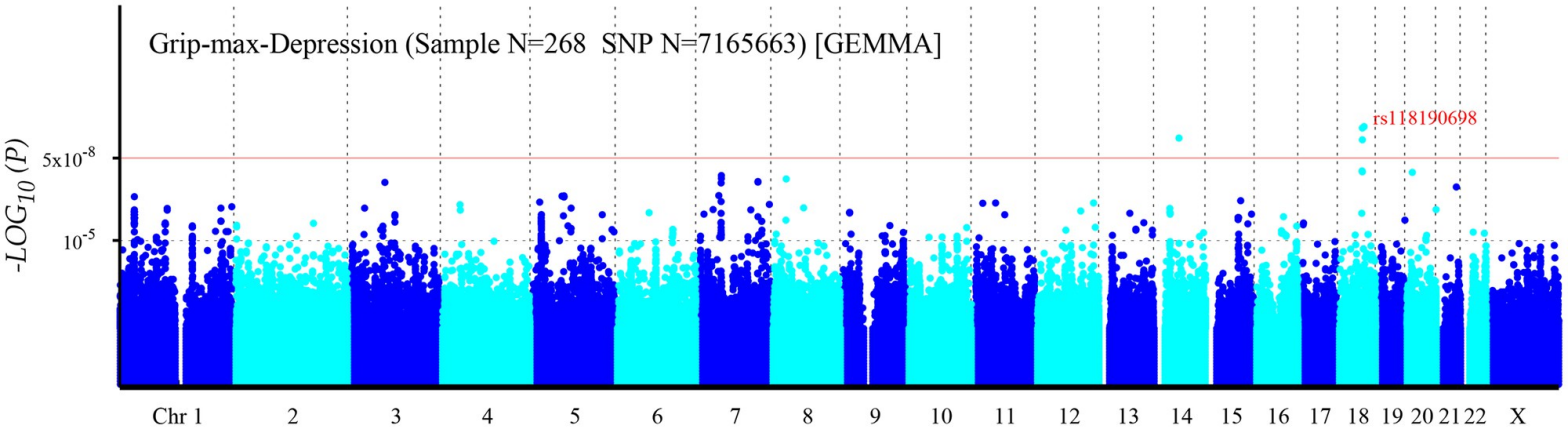
Manhattan plot for bivariate genome-wide association study of grip strength and depression. The horizontal axis represents autosomes and the X chromosome, while the vertical axis represents the *P*-values of SNPs. The red line represents the genome-wide significance threshold (5×10^−8^), and the lower horizontal dashed line represents the suggestive significance level (1×10^−5^).

A bivariate GWAS was performed in 134 DZ twin pairs. As shown in the Q-Q plot (Fig 1) for the bivariate measures of grip strength and depression, the inflation coefficients for the groups had a λ value of 1.027, indicating no stratification of the population. The Manhattan plot (Fig 2) provides a visualization of the results of the GWAS. As shown in the plot, 7 SNPs exceeded the genome-wide significance level (*P*<5×10^−8^) and a total of 336 SNPs reached the level of suggestive significance (*P*<1×10^−5^). The most significant SNP was rs118190698 (*P* = 1.35×10^−9^) located in the *RAB27B* gene on chromosome 18; followed by rs79530590 (*P* = 1.50×10^−9^), located in the *LOC107985152* gene; rs117744620 (*P* = 3.31×10^−9^) located in the *LRR1* gene; rs117546604 (*P* = 3.84×10^−9^), rs150220336 (*P* = 4.39×10^−8^), and rs147079354 (*P* = 4.62×10^−8^), located in the *ME2* gene; and rs79287957 (*P* = 4.62×10^−8^) located close to the *LINC02871* gene. The top 60 SNPs are shown in Table 3 sorted by *P* value.

**Table 3:** Top 60 SNPs that reached P < 1×10^−^5 from bivariate GWAS of grip strength and depression.

SNP	Chr	Band	BP	*P*-value	Gene/Nearest gene
rs118190698	18	q21.2	52480259	1.35E-09	*RAB27B*
rs79530590	18	q21.2	48520209	1.50E-09	*LOC107985152*
rs117744620	14	q21.3	50079611	3.31E-09	*LRR1*
rs117546604	18	q21.2	48472778	3.84E-09	*ME2*
rs150220336	18	q21.2	48388016	4.39E-08	*ME2*
rs147079354	18	q21.2	48447754	4.62E-08	*ME2*
rs79287957	20	p12.2	11048805	4.92E-08	*LINC02871*
rs75534602	7	p12.1	50634883	6.13E-08	*DDC*
rs117533783	7	p12.2	50525408	7.82E-08	*DDC*
rs116994552	8	p21.1	28076758	8.16E-08	*LOC100131127*
rs11973477	7	q32.3	130908819	1.01E-07	*MKLN1*
rs6961574	7	q32.3	130908776	1.04E-07	*MKLN1*
rs149538842	3	p12.3	76440134	1.06E-07	*ROBO2*
rs78161270	7	p12.2	50541930	1.13E-07	*DDC*
rs117549429	21	q22.13	38971496	1.53E-07	*KCNJ6*
rs74214322	7	p13	45401720	3.00E-07	*ELK1P1*
rs118025410	5	q13.2	68859027	3.08E-07	*GTF2H2C*
rs76553625	5	q12.3	64268965	3.19E-07	*CWC27*
rs5773363	1	p35.2	31918631	3.24E-07	*SERINC2*
rs12657552	5	p13	68855113	3.29E-07	*GTF2H2C*
rs4081993	5	p13	68856116	3.33E-07	*GTF2H2C*
rs147869550	15	q23	71596350	4.45E-07	*THSD4*
rs1868887	7	p12.2	50479856	4.76E-07	*IKZF1*
rs79147986	5	p15.1	15593286	5.05E-07	*FBXL7*
rs201380943	11	p11.2	47778625	5.41E-07	*FNBP4*
rs116890548	12	q24.31	121620387	5.43E-07	*P2RX7*
rs573505577	11	p15.1	19918670	5.48E-07	*NAV2*
rs80251137	7	q36.3	155920157	6.09E-07	*LOC105375601*
rs2995920	4	p14.3	37904456	6.24E-07	*TBC1D1*
rs141475535	1	q44	244427543	7.24E-07	*LOC105373262*
rs116961485	8	q13.1	66227114	7.79E-07	*PPIAP86*
rs534143679	3	p22.3	32433754	7.96E-07	*CMTM7*
rs62358249	5	q14.3	84421657	8.02E-07	*RBBP4P6*
rs17008263	1	q41	220877255	8.02E-07	*C1orf115*
rs11851625	14	q12	30314159	8.15E-07	*PRKD1*
rs12563371	1	p21.1	103499755	8.22E-07	*COL11A1*
rs200867673	7	p14.3	32871494	8.88E-07	*DPY19L1P2*
rs2297807	20	q13.33	62575853	8.90E-07	*UCKL1*
rs57042988	7	q31.2	114791544	9.16E-07	*LINC01392*
rs10914394	1	p35.2	31915692	9.18E-07	*LOC105378625*
rs181979988	4	p14.3	38496704	9.38E-07	*LINC01258*
rs141325897	1	p21.1	103598274	9.38E-07	*COL11A1*
rs57244134	12	q22.13	94661218	9.93E-07	*CEP83*
rs10914386	1	p35.2	31909072	1.06E-06	*SERINC2*
rs10914387	1	p35.2	31909124	1.06E-06	*SERINC2*
rs6702129	1	p35.2	31910482	1.06E-06	*SERINC2*
rs1320586	1	p35.2	31908201	1.07E-06	*SERINC2*
rs12122438	1	p35.2	31909448	1.07E-06	*SERINC2*
rs12141959	1	p35.2	31909380	1.07E-06	*SERINC2*
rs6690908	1	p35.2	31910089	1.07E-06	*SERINC2*
rs6675883	1	p35.2	31910202	1.07E-06	*SERINC2*
rs6688664	1	p35.2	31910337	1.07E-06	*SERINC2*
rs139995350	1	p35.2	31910430	1.07E-06	*SERINC2*
rs6691338	1	p35.2	31910655	1.07E-06	*SERINC2*
rs75429043	14	q12	30316306	1.13E-06	*PRKD1*
rs11795332	9	p22.3	15352410	1.13E-06	*RPL7P33*
rs113400337	9	p22.3	15360062	1.13E-06	*RPL7P33*
rs377290438	6	q12	68889736	1.14E-06	*LINC02549*
rs12323862	14	q12	30317951	1.14E-06	*PRKD1*
rs79219406	9	p22.3	15347617	1.18E-06	*RPL7P33*

SNP, nucleotide polymorphism; Chr, chromosome; BP, base pair.

#### Gene-based analysis

In the gene-based analysis, 2 genes reached a significant association level (*P* = 2.36×10^−6^): *GTF2H2C_2* (*P* = 3.08×10^−7^) and *GTF2H2C* (*P* = 3.08×10^−7^). Furthermore, 1,262 genes reached the nominal significance level (*P*<0.05). The top 60 genes are shown in S1 Table, and most of these genes were related to the nervous system, actin, and immune system.

#### Pathway enrichment analysis

In the pathway enrichment analysis, we identified 621 biological pathways associated with grip strength and depression (emp-*P*<0.05). We ranked the top 60 pathways by the strength of association in S2 Table. Most of these pathways were involved in hormone synthesis, the immune system, and the nervous system.

### eQTL analysis

The eQTL analysis across tissues using the Asian population as a reference found that 13 SNPs were significant eQTLs in several tissues, including brain tissues, skeletal muscle, the tibial nerve, and the thyroid (S3 Table; S1–S3 Figs). Among them, the rs10914394 (S1 Fig, Brain–Spinal cord (cervical c-1), *P* value = 5.2×10^−5^, m-value = 1.00; Muscle–Skeletal, *P* value = 4.3×10^−28^, m-value = 1.00; Nerve–Tibial, *P* value = 7.2×10^−13^, m-value = 1.00; Thyroid, *P* value = 4.3×10^−12^, m-value = 1.00), rs10914386 (S2 Fig, Brain–Spinal cord (cervical c-1), *P* value = 1.4×10^−4^, m-value = 1.00; Muscle–Skeletal, *P* value = 1.3×10^−28^, m-value = 1.00; Nerve–Tibial, *P* value = 3.2×10^−13^, m-value = 1.00; Thyroid, *P* value = 1.3×10^−11^, m-value = 1.00), and rs10914387 (S3 Fig, Brain–Spinal cord (cervical c-1), *P* value = 1.4×10^−4^, m-value = 1.00; Muscle–Skeletal, *P* value = 2.5×10^−28^, m-value = 1.00; Nerve–Tibial, *P* value = 3.1×10^−13^, m-value = 1.00; Thyroid, *P* value = 7.2×10^−12^, m-value = 1.00) SNPs were significantly associated with the expression of the *SERINC2* gene in brain tissues and muscle tissues.

### Pleiotropy analysis

Table 4 shows the results of the pleiotropy analysis. In the top 15 SNPs, 9 were associated with both grip strength and depression. In addition, rs117549429 was associated with depression only, and rs79287957, rs75534602, rs117533783, rs149538842, and rs78161270 were associated with grip strength only.

**Table 4:** The results of pleiotropy analysis for bivariate GWAS of grip strength-depression identified top 15 SNPs.

SNP	Chr	BP	*P*-value ^a^	Trait of nonzero *β* ^b^	P for test0 ^c^	P for test1 ^d^	Associated trait
rs118190698	18	52480259	1.35E-09	G; D	1.88E-06	2.25E-04	G; D
rs79530590	18	48520209	1.50E-09	G; D	5.52E-06	2.09E-03	G; D
rs117744620	14	50079611	3.31E-09	G; D	1.11E-07	7.75E-03	G; D
rs117546604	18	48472778	3.84E-09	G; D	1.70E-06	4.19E-04	G; D
rs150220336	18	48388016	4.39E-08	G; D	9.39E-07	1.77E-04	G; D
rs147079354	18	48447754	4.62E-08	G; D	1.00E-06	1.66E-04	G; D
rs79287957	20	11048805	4.92E-08	G	3.10E-05	5.61E-01	G
rs75534602	7	50634883	6.13E-08	G	3.56E-05	2.45E-01	G
rs117533783	7	50525408	7.82E-08	G	2.10E-05	9.59E-02	G
rs116994552	8	28076758	8.16E-08	G; D	1.03E-06	2.48E-02	G; D
rs11973477	7	130908819	1.01E-07	G; D	2.03E-06	1.06E-03	G; D
rs6961574	7	130908776	1.04E-07	G; D	1.97E-06	1.03E-03	G; D
rs149538842	3	76440134	1.06E-07	G	5.63E-06	2.60E-01	G
rs78161270	7	50541930	1.13E-07	G	2.86E-06	1.20E-01	G
rs117549429	21	38971496	1.53E-07	D	1.87E-02	5.90E-02	D

SNP, nucleotide polymorphism; Chr, chromosome; BP, base pair; G, grip strength; D, depression.

^a^ The *P*-value was derived from bivariate GWAS.

^b^ Sequential tests of pleiotropy with a *P* threshold of 0.05.

^c^ Single test of the number of traits associated with genotype, H0 (test0): all betas = 0.

^d^ Single test of the number of traits associated with genotype, H0 (test1): one or less beta is nonzero.

## Discussion

A total of 369 pairs of twins were included in this study. The genetic correlation between grip strength and depression was evaluated by a bivariate genetic model. It was found that there was a moderate genetic correlation between grip strength and depression, and the genetic correlation coefficient was -0.41 (-0.96, -0.15), suggesting that there was a common genetic basis between them. At present, most of the studies on the heritability of grip strength and depression are carried out for a single variable. Previous studies have shown that the heritability of grip strength and depression is high, but there is still a gap in the research in determining their common genetic correlation coefficient.

Then, a bivariate GWAS was carried out in 134 pairs of DZ twins to identify the common SNPs, genes, and pathways of grip strength and depression. In the SNP-based analysis, rs117744620, located in the *LRR1* gene on chromosome 14, exceeded the genome-wide significance level. *LRR1* encodes a protein with a leucine-rich repeat. A previous study showed that *LRR1* regulates 4-1BB-mediated signaling cascades, which activate NF-кB [47], and NF-кB could affect depression. *LRR1* can also affect actin [48], thereby affecting muscle strength. Three SNPs (rs117546604, rs150220336, rs147079354) located in or near the *ME2* gene also exceeded the genome-wide significance level. The *ME2* gene encodes a mitochondrial NAD-dependent malic enzyme. *ME2* has been shown to be associated with generalized epilepsy [49], suggesting that *ME2* may affect both the nervous system and muscle strength. In addition, *ME2* has been found to be associated with susceptibility to psychosis and mania [50], and the knockdown of *ME2* may affect PI3K/AKT signaling [51], all suggesting that *ME2* is also associated with depression.

In the top 60 SNPs that reached the suggested level of association, three SNPs (rs75534602, rs117533783, rs78161270) were located near the *DDC* gene. A study has shown that the *DDC* gene is associated with postpartum anxiety [52]. In addition, the *DDC* gene was also associated with aromatic L-amino acid decarboxylase deficiency (AADCD), a neurotransmitter metabolism disorder in the mainland Chinese population, the main symptoms of which include early-onset hypotonia [53]. Therefore, the *DDC* gene may affect grip strength and depression simultaneously. rs116890548 is located in the *P2RX7* gene on chromosome 12. *P2RX7* is associated with the inflammatory response and neuroimmune mechanisms of depression and neurodegenerative diseases [54, 55]. This gene can also affect calcium channels [56, 57] and is associated with bone and joint diseases [58]. Therefore, *P2RX7* may affect muscle strength. Moreover, three SNPs (rs11851625, rs75429043, rs12323862) were located in the *PRKD1* gene on chromosome 14. The *PRKD1* gene can regulate actin [59] and affect skeletal muscle [60]. The *PRKD1* gene is also associated with neurons [61], NF-кB [62], and the inflammatory response [63]. Therefore, *PRKD1* may also regulate depression and muscle strength simultaneously.

In gene-based analysis, we found 2 genes that exhibited a significant association, *GTF2H2C_2* and *GTF2H2C*. However, there are too few studies on these two genes, and we cannot yet explain the relationship of these two genes with the phenotype. Furthermore, among the genes with nominal significance levels, many were associated with grip strength and depression: (1) *FNBP1* can affect neuronal dendrites [64] and actin [65], which may affect grip strength and depression; (2) *MOG* is associated with multiple sclerosis [66, 67], the main symptoms of which include depression and decreased muscle strength; (3) the *SACS* gene can affect motor and sensory neuropathy [68], and is associated with complex neurological disorders [69]; (4) the *KMO* gene is associated with depression [70, 71] and Huntington’s disease [72], the symptoms of which include muscle atrophy; (5) the *TECPR2* gene is tightly linked to the nervous system [73–76] and may influence depression and grip strength by affecting nerves; and (6) *MGTA5* can affect the severity of multiple sclerosis [77, 78]. *MGTA5* may also be associated with attempted suicide [79], and therefore may be associated with depression. In addition, the expression levels of KCNN4 [80, 81], MOG [82, 83], and SNHG12 [84, 85] genes can influence inflammatory factors, which may affect grip strength and depression by influencing the inflammatory response.

In the pathway enrichment analysis, many pathways were related to hormone synthesis and neural signal transduction. (1) Androgen biosynthesis, steroid hormones, and steroid hormone biosynthesis are related to androgen synthesis and metabolism. Androgen levels are not only associated with depression but also affect grip strength [86, 87]. (2) Potassium channels and the nervous system can regulate the resting membrane potential in neurons, and it can affect both depression and muscle strength [88, 89]. (3) Signaling by RHO GTPases can affect guanine nucleotides, and studies have shown that RHO GTPases can affect actin [90] and neuronal development [91, 92], thereby affecting muscle strength and depression. (4) The phospholipase C mediated cascade, activated point mutants of *FGFR2*, and *FGFR* ligand binding and activation are related to fibroblast growth factor receptor. Fibroblast growth factors are associated with skeletal muscle development [93], and a study has found that fibroblast growth factor-2 can affect depression [94]. (5) Cytokine receptor interaction pathway can affect cytokines. Cytokines are soluble extracellular proteins or glycoproteins that are crucial intercellular regulators and mobilizers of cells engaged in innate as well as adaptive inflammatory host defenses. This pathway may affect grip strength and depression by influencing the inflammatory response.

The current research has several strengths. First, this is the first genetic correlation and bivariate GWAS on grip strength and depression, which will help to study the common genetic basis of these phenotypes. Second, our study was conducted in a twin sample, which is more effective when exploring complex phenotypes such as depression [32]. In addition, we performed a pleiotropic analysis, and the results showed that a number of the SNPs we obtained were indeed associated with both grip strength and depression, which further confirmed the accuracy of our study. At the same time, our research has some limitations. First, because it is difficult to recruit twin samples, our sample size was relatively small, which limited our ability to find more potential SNPs, genes, and pathways. Smaller sample sizes may also lead to lower power, but we have used pleiotropy analysis to improve the credibility of the results. Second, we cannot fully explain the SNP, gene, and pathway associations that we found. For example, although two genes exceeded the Bonferroni-corrected significance threshold and the same genes have also been found in univariate GWASs, we cannot explain the associated biological mechanism. Third, because our sample included both middle-aged and older adults, and the depression questionnaire we used was the GDS-30, this may have had an impact on the assessment of depressive symptoms.

## Conclusion

In conclusion, this bivariate GWAS identified potentially common pleiotropic SNPs, genes, and pathways in grip strength and depression. However, more in-depth studies are still needed to validate our results.

## Supporting information

S1 Table Top 60 genes associated with grip strength-depression from gene-based analysis. (DOCX)

S2 Table The top 60 pathways associated with grip strength-depression from pathway enrichment analysis. (DOCX)

S3 Table eQTL of grip strength and depression. (DOCX)

S1 Fig Expression quantitative trait loci (eQTL) analysis of rs10914394 with SERINC2 across tissue types from the GTEx database. NES is the normalized effect size (β) from single-tissue eQTL analysis. P-value is from the t-test that compares observed NES in single-tissue eQTL analysis to the null hypothesis of no NES. m-value represents a posterior probability that the effect of eQTL exists in each tissue of a cross-tissue meta-analysis.
(TIF)

S2 Fig Expression quantitative trait loci (eQTL) analysis of rs10914386 with SERINC2 across tissue types from the GTEx database. NES is the normalized effect size (β) from single-tissue eQTL analysis. P-value is from the t-test that compares observed NES in single-tissue eQTL analysis to the null hypothesis of no NES. m-value represents a posterior probability that the effect of eQTL exists in each tissue of a cross-tissue meta-analysis.
(TIF)

S3 Fig Expression quantitative trait loci (eQTL) analysis of rs10914387 with SERINC2 across tissue types from the GTEx database. NES is the normalized effect size (β) from single-tissue eQTL analysis. P-value is from the t-test that compares observed NES in single-tissue eQTL analysis to the null hypothesis of no NES. m-value represents a posterior probability that the effect of eQTL exists in each tissue of a cross-tissue meta-analysis.
(TIF)
