# Supplementary material for: A genetic correlation and bivariate genome-wide association study of grip strength and depression
Source: PLoS One. 2022 Dec 15;17(12):e0278392. doi: 10.1371/journal.pone.0278392 (PMC9754196; doi:10.1371/journal.pone.0278392)
Supplement: S1 Table — (DOCX) [file pone.0278392.s001.docx]

**S1 Table.** Top 60 genes associated with grip strength-depression from gene-based analysis.

| Gene | Chr | SNP (N) | Start | Stop | *P*-value | Top-SNP | Top-SNP *P*-value |
| --- | --- | --- | --- | --- | --- | --- | --- |
| *GTF2H2C_2* | 5 | 2 | 68856073 | 68888352 | 1.00E-06 | rs118025410 | 3.08E-07 |
| *GTF2H2C* | 5 | 2 | 68856050 | 68888729 | 1.00E-06 | rs118025410 | 3.08E-07 |
| *TMEM257* | 23 | 3 | 144908927 | 144911370 | 2.00E-05 | rs5919909 | 1.48E-05 |
| *OR6N1* | 1 | 6 | 158735533 | 158736472 | 2.40E-05 | rs857826 | 1.90E-05 |
| *TRIM43* | 2 | 4 | 96257765 | 96265469 | 4.70E-05 | rs147308550 | 1.97E-05 |
| *UCKL1* | 20 | 10 | 62571181 | 62587800 | 6.00E-05 | rs2297807 | 8.90E-07 |
| *C2orf70* | 2 | 29 | 26785480 | 26802395 | 8.20E-05 | rs935170 | 1.24E-05 |
| *FNBP1* | 9 | 225 | 132649465 | 132805473 | 8.90E-05 | rs112586081 | 9.94E-06 |
| *MIR4427* | 1 | 2 | 233759897 | 233759965 | 1.00E-04 | rs701213 | 6.37E-04 |
| *OR6K6* | 1 | 2 | 158724605 | 158725637 | 1.21E-04 | rs16841001 | 2.24E-05 |
| *MCF2L-AS1* | 13 | 3 | 113621797 | 113622952 | 1.58E-04 | rs12323211 | 2.67E-03 |
| *TPP1* | 11 | 7 | 6633996 | 6640692 | 1.73E-04 | rs1800723 | 1.67E-05 |
| *TFCP2L1* | 2 | 130 | 121974163 | 122042778 | 1.82E-04 | rs78093243 | 2.18E-04 |
| *KCNN4* | 19 | 40 | 44270684 | 44285409 | 2.13E-04 | rs563995 | 4.54E-04 |
| *MOG* | 6 | 4 | 29624757 | 29640149 | 2.25E-04 | rs1984840 | 5.61E-04 |
| *OR51I1* | 11 | 7 | 5461771 | 5462783 | 2.27E-04 | rs16930982 | 1.95E-04 |
| *IRGC* | 19 | 2 | 44220213 | 44224169 | 2.40E-04 | rs11555891 | 1.82E-04 |
| *MED23* | 6 | 28 | 131895105 | 131949379 | 2.49E-04 | rs2246012 | 4.56E-05 |
| *SNHG12* | 1 | 2 | 28905049 | 28908366 | 2.61E-04 | rs1342086 | 2.38E-04 |
| *ZCCHC17* | 1 | 151 | 31769828 | 31837800 | 2.99E-04 | rs756233 | 1.12E-04 |
| *HCG4B* | 6 | 8 | 29892368 | 29894992 | 3.40E-04 | rs9259814 | 3.47E-04 |
| *CNIH3* | 1 | 427 | 224804178 | 224928249 | 4.12E-04 | rs12564263 | 2.70E-05 |
| *COMMD10* | 5 | 937 | 115420726 | 115628978 | 4.32E-04 | rs7720507 | 9.93E-05 |
| *SACS* | 13 | 232 | 23902961 | 24007867 | 4.35E-04 | rs9550964 | 6.71E-06 |
| *TOX4* | 14 | 55 | 21945334 | 21967319 | 4.42E-04 | rs933192 | 5.20E-05 |
| *CYP17A1* | 10 | 16 | 104590287 | 104597290 | 5.02E-04 | rs743572 | 1.22E-04 |
| *TNFRSF6B* | 20 | 7 | 62328003 | 62330051 | 5.27E-04 | rs2257440 | 7.20E-04 |
| *C10orf32* | 10 | 20 | 104613966 | 104624718 | 5.43E-04 | rs144499417 | 4.10E-05 |
| *DGKE* | 17 | 67 | 54911459 | 54946036 | 5.83E-04 | rs4365350 | 3.74E-04 |
| *OGN* | 9 | 39 | 95146248 | 95166937 | 6.14E-04 | rs113642084 | 1.32E-04 |
| *DOPEY1* | 6 | 158 | 83777384 | 83878190 | 6.22E-04 | rs495944 | 2.07E-05 |
| *WBP1L* | 10 | 182 | 104503726 | 104576021 | 6.38E-04 | rs284857 | 7.22E-06 |
| *SERINC2* | 1 | 95 | 31882411 | 31907527 | 6.66E-04 | rs4233 | 1.30E-06 |
| *RRP8* | 11 | 8 | 6621143 | 6624880 | 6.67E-04 | rs12420389 | 6.15E-04 |
| *PGM3* | 6 | 34 | 83874592 | 83903655 | 6.74E-04 | rs522659 | 1.13E-05 |
| *TIMM8A* | 23 | 5 | 100600643 | 100603957 | 7.39E-04 | rs3027649 | 3.30E-05 |
| *KMO* | 1 | 169 | 241695433 | 241758949 | 7.72E-04 | rs2050504 | 1.89E-05 |
| *RTEL1* | 20 | 95 | 62289162 | 62327606 | 7.96E-04 | rs2236507 | 4.12E-04 |
| *WDR81* | 17 | 22 | 1619816 | 1641893 | 8.12E-04 | rs1045806 | 4.57E-04 |
| *ARFRP1* | 20 | 12 | 62329994 | 62339365 | 8.53E-04 | rs1291209 | 2.47E-04 |
| *MIR548AA1* | 1 | 45 | 100154610 | 100178513 | 8.81E-04 | rs12136609 | 2.92E-04 |
| *MIR548D1* | 1 | 45 | 100154610 | 100178513 | 9.13E-04 | rs12136609 | 2.92E-04 |
| *ME1* | 6 | 486 | 83920109 | 84140938 | 9.60E-04 | rs1180183 | 1.26E-04 |
| *BANP* | 16 | 503 | 87985037 | 88110924 | 9.70E-04 | rs11646420 | 3.72E-04 |
| *NARS* | 18 | 45 | 55267893 | 55289177 | 9.72E-04 | rs112490571 | 1.82E-04 |
| *C1QTNF4* | 11 | 2 | 47611215 | 47615961 | 9.80E-04 | rs140490588 | 4.80E-04 |
| *OMD* | 9 | 14 | 95176526 | 95186836 | 9.90E-04 | rs34860658 | 3.27E-04 |
| *SLITRK2* | 23 | 6 | 144899346 | 144907360 | 1.00E-03 | rs7053492 | 2.03E-04 |
| *CDH8* | 16 | 695 | 61685914 | 62070739 | 1.01E-03 | rs4567699 | 6.00E-04 |
| *LDB1* | 10 | 7 | 103867324 | 103880210 | 1.03E-03 | rs3781296 | 4.16E-04 |
| *CENPP* | 9 | 518 | 95087749 | 95377437 | 1.08E-03 | rs72754487 | 1.32E-04 |
| *TAF1B* | 2 | 354 | 9983570 | 10074545 | 1.16E-03 | rs2303921 | 1.30E-04 |
| *TECPR2* | 14 | 365 | 102829299 | 102968818 | 1.16E-03 | rs115563083 | 4.41E-04 |
| *TNRC18* | 7 | 280 | 5346422 | 5463177 | 1.21E-03 | rs6463418 | 7.18E-06 |
| *SSTR4* | 20 | 4 | 23016056 | 23017314 | 1.21E-03 | rs3746726 | 1.34E-03 |
| *ATP8B5P* | 9 | 90 | 35406751 | 35483026 | 1.23E-03 | rs10972468 | 2.67E-05 |
| *ZGPAT* | 20 | 55 | 62338793 | 62367494 | 1.24E-03 | rs6062501 | 4.07E-04 |
| *MGAT5* | 2 | 518 | 135011829 | 135212192 | 1.25E-03 | rs7566254 | 3.24E-04 |
| *NOL8* | 9 | 52 | 95059639 | 95087876 | 1.29E-03 | rs7872423 | 3.27E-04 |
| *MIR22HG* | 17 | 4 | 1614797 | 1619566 | 1.32E-03 | rs4790812 | 1.18E-03 |

Chr, chromosome; SNP, nucleotide polymorphism.
