# Supplementary material for: A genetic correlation and bivariate genome-wide association study of grip strength and depression
Source: PLoS One. 2022 Dec 15;17(12):e0278392. doi: 10.1371/journal.pone.0278392 (PMC9754196; doi:10.1371/journal.pone.0278392)
Supplement: S2 Table — (DOCX) [file pone.0278392.s002.docx]

**S2 Table.** The top 60 pathways associated with grip strength-depression from pathway enrichment analysis.

| Pathway | chisq-*P* | emp-*P* |
| --- | --- | --- |
| KEGG_PPAR_SIGNALING_PATHWAY | 3.55E-04 | 1.02E-04 |
| REACTOME_METABOLISM_OF_MRNA | 5.53E-04 | 2.79E-04 |
| REACTOME_METABOLISM_OF_RNA | 6.37E-04 | 3.83E-04 |
| REACTOME_NONSENSE_MEDIATED_DECAY_ENHANCED_BY_THE_EXON_JUNCTION_COMPLEX | 1.04E-03 | 5.30E-04 |
| KEGG_GLYCEROPHOSPHOLIPID_METABOLISM | 3.59E-03 | 6.40E-04 |
| REACTOME_REGULATION_OF_WATER_BALANCE_BY_RENAL_AQUAPORINS | 2.18E-03 | 8.40E-04 |
| REACTOME_ANDROGEN_BIOSYNTHESIS | 1.13E-03 | 1.14E-03 |
| REACTOME_ENDOGENOUS_STEROLS | 1.13E-03 | 1.14E-03 |
| REACTOME_CLASS_I_MHC_MEDIATED_ANTIGEN_PROCESSING_PRESENTATION | 5.01E-03 | 1.22E-03 |
| REACTOME_LATE_PHASE_OF_HIV_LIFE_CYCLE | 2.83E-03 | 1.33E-03 |
| REACTOME_HIV_LIFE_CYCLE | 2.83E-03 | 1.34E-03 |
| REACTOME_STEROID_HORMONES | 3.98E-03 | 1.43E-03 |
| REACTOME_POTASSIUM_CHANNELS | 4.40E-03 | 1.50E-03 |
| REACTOME_METABOLISM_OF_STEROID_HORMONES_AND_VITAMINS_A_AND_D | 5.79E-03 | 1.75E-03 |
| KEGG_STARCH_AND_SUCROSE_METABOLISM | 2.02E-03 | 2.12E-03 |
| REACTOME_TRANSPORT_TO_THE_GOLGI_AND_SUBSEQUENT_MODIFICATION | 1.81E-03 | 2.17E-03 |
| KEGG_CIRCADIAN_RHYTHM_MAMMAL | 2.01E-03 | 2.19E-03 |
| KEGG_CYTOKINE_CYTOKINE_RECEPTOR_INTERACTION | 1.97E-03 | 2.22E-03 |
| KEGG_RIBOFLAVIN_METABOLISM | 2.02E-03 | 2.28E-03 |
| REACTOME_METABOLISM_OF_LIPIDS_AND_LIPOPROTEINS | 4.01E-03 | 2.38E-03 |
| REACTOME_PHOSPHOLIPASE_C_MEDIATED_CASCADE | 2.62E-03 | 2.44E-03 |
| REACTOME_SIGNALING_BY_RHO_GTPASES | 4.40E-03 | 2.49E-03 |
| REACTOME_SHC_MEDIATED_CASCADE | 2.62E-03 | 2.50E-03 |
| REACTOME_ACTIVATED_POINT_MUTANTS_OF_FGFR2 | 2.62E-03 | 2.56E-03 |
| REACTOME_PI_3K_CASCADE | 2.62E-03 | 2.56E-03 |
| REACTOME_FGFR_LIGAND_BINDING_AND_ACTIVATION | 2.62E-03 | 2.56E-03 |
| BIOCARTA_PPARA_PATHWAY | 3.79E-03 | 2.58E-03 |
| REACTOME_PI3K_CASCADE | 2.62E-03 | 2.59E-03 |
| KEGG_STEROID_HORMONE_BIOSYNTHESIS | 4.91E-03 | 2.65E-03 |
| REACTOME_SIGNALING_BY_FGFR_MUTANTS | 2.62E-03 | 2.67E-03 |
| REACTOME_NEGATIVE_REGULATION_OF_FGFR_SIGNALING | 2.62E-03 | 2.70E-03 |
| KEGG_COMPLEMENT_AND_COAGULATION_CASCADES | 2.99E-03 | 3.09E-03 |
| REACTOME_TRANSPORT_OF_RIBONUCLEOPROTEINS_INTO_THE_HOST_NUCLEUS | 3.46E-03 | 3.30E-03 |
| REACTOME_GLUCOSE_TRANSPORT | 3.46E-03 | 3.32E-03 |
| REACTOME_REGULATION_OF_GLUCOKINASE_BY_GLUCOKINASE_REGULATORY_PROTEIN | 3.46E-03 | 3.32E-03 |
| REACTOME_FATTY_ACID_TRIACYLGLYCEROL_AND_KETONE_BODY_METABOLISM | 6.01E-03 | 3.44E-03 |
| REACTOME_NEP_NS2_INTERACTS_WITH_THE_CELLULAR_EXPORT_MACHINERY | 3.46E-03 | 3.50E-03 |
| KEGG_NICOTINATE_AND_NICOTINAMIDE_METABOLISM | 4.31E-03 | 3.56E-03 |
| REACTOME_METABOLISM_OF_NON_CODING_RNA | 3.46E-03 | 3.61E-03 |
| KEGG_PENTOSE_PHOSPHATE_PATHWAY | 2.83E-03 | 3.76E-03 |
| REACTOME_NEURONAL_SYSTEM | 1.18E-02 | 4.08E-03 |
| KEGG_BASAL_CELL_CARCINOMA | 5.28E-03 | 4.10E-03 |
| REACTOME_CLASS_A1_RHODOPSIN_LIKE_RECEPTORS | 6.23E-03 | 4.11E-03 |
| KEGG_RNA_DEGRADATION | 4.58E-03 | 4.17E-03 |
| REACTOME_PEPTIDE_LIGAND_BINDING_RECEPTORS | 6.04E-03 | 4.22E-03 |
| REACTOME_AQUAPORIN_MEDIATED_TRANSPORT | 6.40E-03 | 4.31E-03 |
| REACTOME_RNA_POL_I_TRANSCRIPTION_INITIATION | 5.58E-03 | 4.65E-03 |
| REACTOME_N_GLYCAN_ANTENNAE_ELONGATION_IN_THE_MEDIAL_TRANS_GOLGI | 3.06E-03 | 4.90E-03 |
| REACTOME_BIOSYNTHESIS_OF_THE_N_GLYCAN_PRECURSOR_DOLICHOL_LIPID_LINKED_OLIGOSACCHARIDE_LLO_AND_TRANSFER_TO_A_NASCENT_PROTEIN | 5.05E-03 | 4.90E-03 |
| REACTOME_RNA_POL_I_TRANSCRIPTION_TERMINATION | 5.58E-03 | 5.02E-03 |
| REACTOME_N_GLYCAN_ANTENNAE_ELONGATION | 3.06E-03 | 5.11E-03 |
| REACTOME_RNA_POL_I_TRANSCRIPTION | 5.58E-03 | 5.11E-03 |
| REACTOME_SYNTHESIS_OF_SUBSTRATES_IN_N_GLYCAN_BIOSYTHESIS | 5.05E-03 | 5.37E-03 |
| BIOCARTA_KREB_PATHWAY | 5.70E-03 | 5.72E-03 |
| REACTOME_HIV_INFECTION | 7.02E-03 | 5.84E-03 |
| KEGG_UBIQUITIN_MEDIATED_PROTEOLYSIS | 4.99E-03 | 5.95E-03 |
| REACTOME_PHASE1_FUNCTIONALIZATION_OF_COMPOUNDS | 1.56E-02 | 5.98E-03 |
| REACTOME_CRMPS_IN_SEMA3A_SIGNALING | 6.28E-03 | 6.01E-03 |
| REACTOME_CYTOCHROME_P450_ARRANGED_BY_SUBSTRATE_TYPE | 1.62E-02 | 6.02E-03 |
| KEGG_PPAR_SIGNALING_PATHWAY | 3.55E-04 | 1.02E-04 |

emp-*P*, empirical *p*-value.
