# Supplementary material for: A genetic correlation and bivariate genome-wide association study of grip strength and depression
Source: PLoS One. 2022 Dec 15;17(12):e0278392. doi: 10.1371/journal.pone.0278392 (PMC9754196; doi:10.1371/journal.pone.0278392)
Supplement: S3 Table — (DOCX) [file pone.0278392.s003.docx]

**S3 Table.** eQTL of grip strength and depression.

| SNPs | Tissue | Gene | *P*-value |
| --- | --- | --- | --- |
| rs17008263 | Brain - Nucleus accumbens (basal ganglia) | *C1orf115* | 3.30E-17 |
|  | Nerve - Tibial | *C1orf115* | 1.90E-13 |
|  | Lung | *C1orf115* | 3.50E-08 |
|  | Brain - Putamen (basal ganglia) | *C1orf115* | 4.10E-07 |
| rs10914394 | Muscle - Skeletal | *SERINC2* | 4.30E-28 |
|  | Testis | *SERINC2* | 2.10E-14 |
|  | Nerve - Tibial | *SERINC2* | 7.20E-13 |
|  | Thyroid | *SERINC2* | 4.30E-12 |
|  | Thyroid | *FABP3* | 6.80E-07 |
|  | Muscle - Skeletal | *FABP3* | 8.60E-05 |
| rs10914386 | Muscle - Skeletal | *SERINC2* | 1.30E-28 |
|  | Testis | *SERINC2* | 1.20E-15 |
|  | Nerve - Tibial | *SERINC2* | 3.20E-13 |
|  | Thyroid | *SERINC2* | 1.30E-11 |
|  | Thyroid | *FABP3* | 1.60E-06 |
| rs10914387 | Muscle - Skeletal | *SERINC2* | 2.50E-28 |
|  | Testis | *SERINC2* | 1.60E-15 |
|  | Nerve - Tibial | *SERINC2* | 3.10E-13 |
|  | Thyroid | *SERINC2* | 7.30E-12 |
|  | Thyroid | *FABP3* | 1.50E-06 |
|  | Muscle - Skeletal | *FABP3* | 4.30E-05 |
| rs6702129 | Muscle - Skeletal | *SERINC2* | 3.10E-34 |
|  | Testis | *SERINC2* | 4.70E-18 |
|  | Nerve - Tibial | *SERINC2* | 3.10E-15 |
|  | Thyroid | *SERINC2* | 1.70E-12 |
|  | Thyroid | *FABP3* | 2.20E-08 |
|  | Kidney - Cortex | *LINC01225* | 2.80E-06 |
|  | Muscle - Skeletal | *NKAIN1* | 4.20E-05 |
|  | Muscle - Skeletal | *FABP3* | 5.90E-05 |
| rs1320586 | Muscle - Skeletal | *SERINC2* | 5.20E-35 |
|  | Testis | *SERINC2* | 2.30E-18 |
|  | Nerve - Tibial | *SERINC2* | 2.80E-14 |
|  | Thyroid | *SERINC2* | 2.60E-12 |
|  | Kidney - Cortex | *LINC01226* | 3.80E-10 |
|  | Thyroid | *FABP3* | 2.60E-08 |
|  | Muscle - Skeletal | *FABP3* | 3.20E-05 |
|  | Muscle - Skeletal | *NKAIN1* | 4.10E-05 |
| rs12122438 | Muscle - Skeletal | *SERINC2* | 8.20E-29 |
|  | Testis | *SERINC2* | 5.90E-15 |
|  | Nerve - Tibial | *SERINC2* | 1.30E-12 |
|  | Thyroid | *SERINC2* | 8.60E-12 |
|  | Thyroid | *FABP3* | 1.50E-06 |
|  | Muscle - Skeletal | *FABP3* | 1.10E-04 |
| rs12141959 | Muscle - Skeletal | *SERINC2* | 8.30E-35 |
|  | Testis | *SERINC2* | 3.80E-18 |
|  | Nerve - Tibial | *SERINC2* | 2.40E-14 |
|  | Thyroid | *SERINC2* | 2.60E-12 |
|  | Thyroid | *FABP3* | 2.60E-08 |
|  | Kidney - Cortex | *LINC01225* | 2.80E-06 |
|  | Muscle - Skeletal | *FABP3* | 2.80E-05 |
|  | Muscle - Skeletal | *NKAIN1* | 3.90E-05 |
| rs6690908 | Muscle - Skeletal | *SERINC2* | 1.00E-28 |
|  | Testis | *SERINC2* | 1.60E-15 |
|  | Nerve - Tibial | *SERINC2* | 3.10E-13 |
|  | Thyroid | *SERINC2* | 7.30E-12 |
|  | Thyroid | *FABP3* | 1.50E-06 |
|  | Muscle - Skeletal | *FABP3* | 4.60E-05 |
| rs6675883 | Muscle - Skeletal | *SERINC2* | 8.20E-29 |
|  | Testis | *SERINC2* | 5.90E-15 |
|  | Nerve - Tibial | *SERINC2* | 1.30E-12 |
|  | Thyroid | *SERINC2* | 8.60E-12 |
|  | Thyroid | *FABP3* | 1.50E-06 |
|  | Muscle - Skeletal | *FABP3* | 1.10E-04 |
| rs6688664 | Muscle - Skeletal | *SERINC2* | 8.20E-29 |
|  | Testis | *SERINC2* | 5.90E-15 |
|  | Nerve - Tibial | *SERINC2* | 1.30E-12 |
|  | Thyroid | *SERINC2* | 8.60E-12 |
|  | Cells - Cultured fibroblasts | *SERINC2* | 5.50E-10 |
|  | Thyroid | *FABP3* | 1.50E-06 |
|  | Muscle - Skeletal | *FABP3* | 1.10E-04 |
| rs139995350 | Muscle - Skeletal | *SERINC2* | 1.00E-28 |
|  | Testis | *SERINC2* | 1.60E-15 |
|  | Nerve - Tibial | *SERINC2* | 3.10E-13 |
|  | Thyroid | *SERINC2* | 7.30E-12 |
|  | Thyroid | *FABP3* | 1.50E-06 |
|  | Muscle - Skeletal | *FABP3* | 4.60E-05 |
| rs6691338 | Muscle - Skeletal | *SERINC2* | 1.00E-28 |
|  | Testis | *SERINC2* | 1.60E-15 |
|  | Nerve - Tibial | *SERINC2* | 3.10E-13 |
|  | Thyroid | *SERINC2* | 7.30E-12 |
|  | Thyroid | *FABP3* | 1.50E-06 |
|  | Muscle - Skeletal | *FABP3* | 4.60E-05 |
