# Supplementary figures and images for: A genetic correlation and bivariate genome-wide association study of grip strength and depression
Source: PLoS One. 2022 Dec 15;17(12):e0278392. doi: 10.1371/journal.pone.0278392 (PMC9754196; doi:10.1371/journal.pone.0278392)

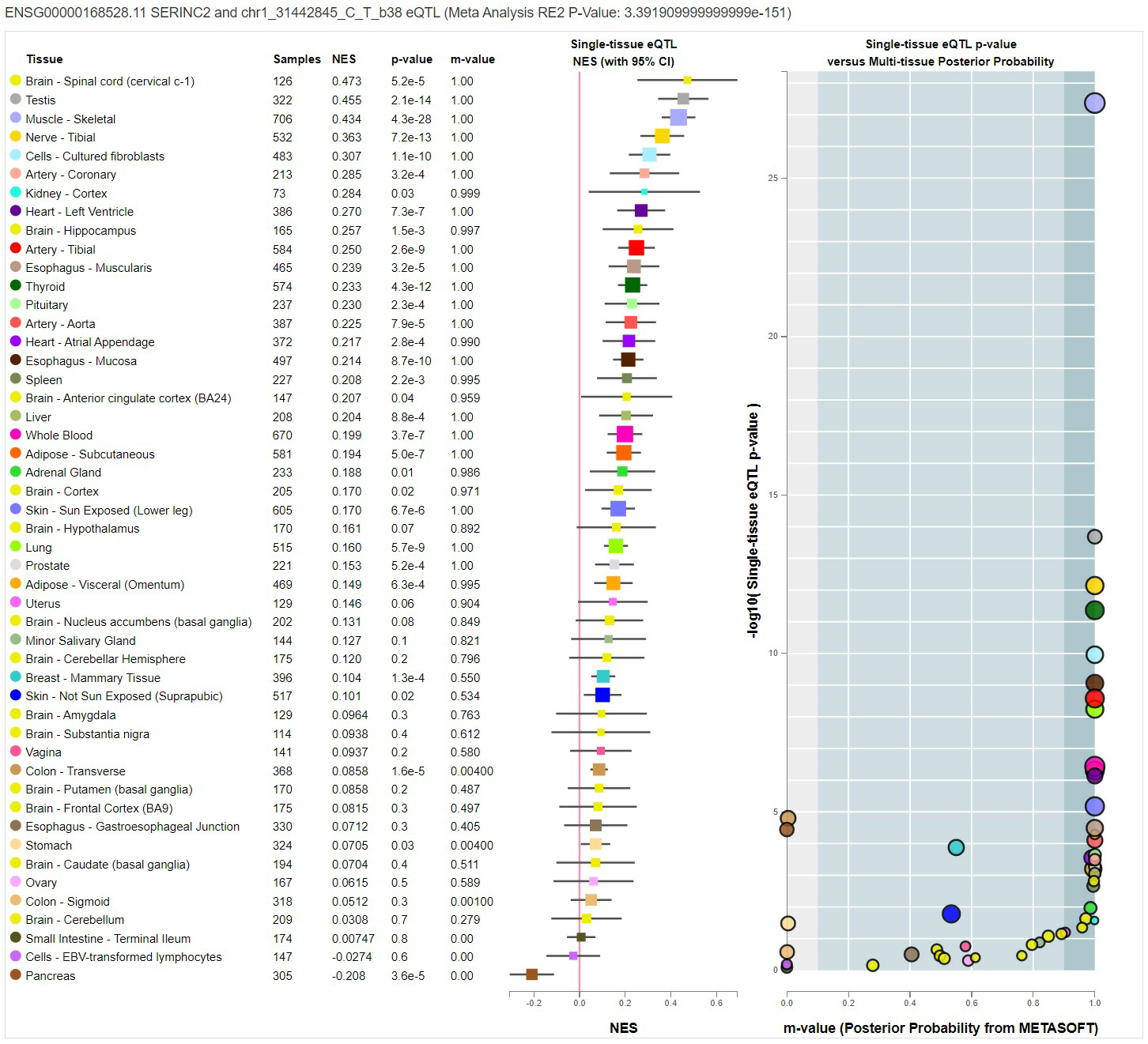

Supplement: S1 Fig — NES is the normalized effect size (β) from single-tissue eQTL analysis. P-value is from the t-test that compares observed NES in single-tissue eQTL analysis to the null hypothesis of no NES. m-value represents a posterior probability that the effect of eQTL exists in each tissue of a cross-tissue meta-analysis. (TIF) [file pone.0278392.s004.tif]

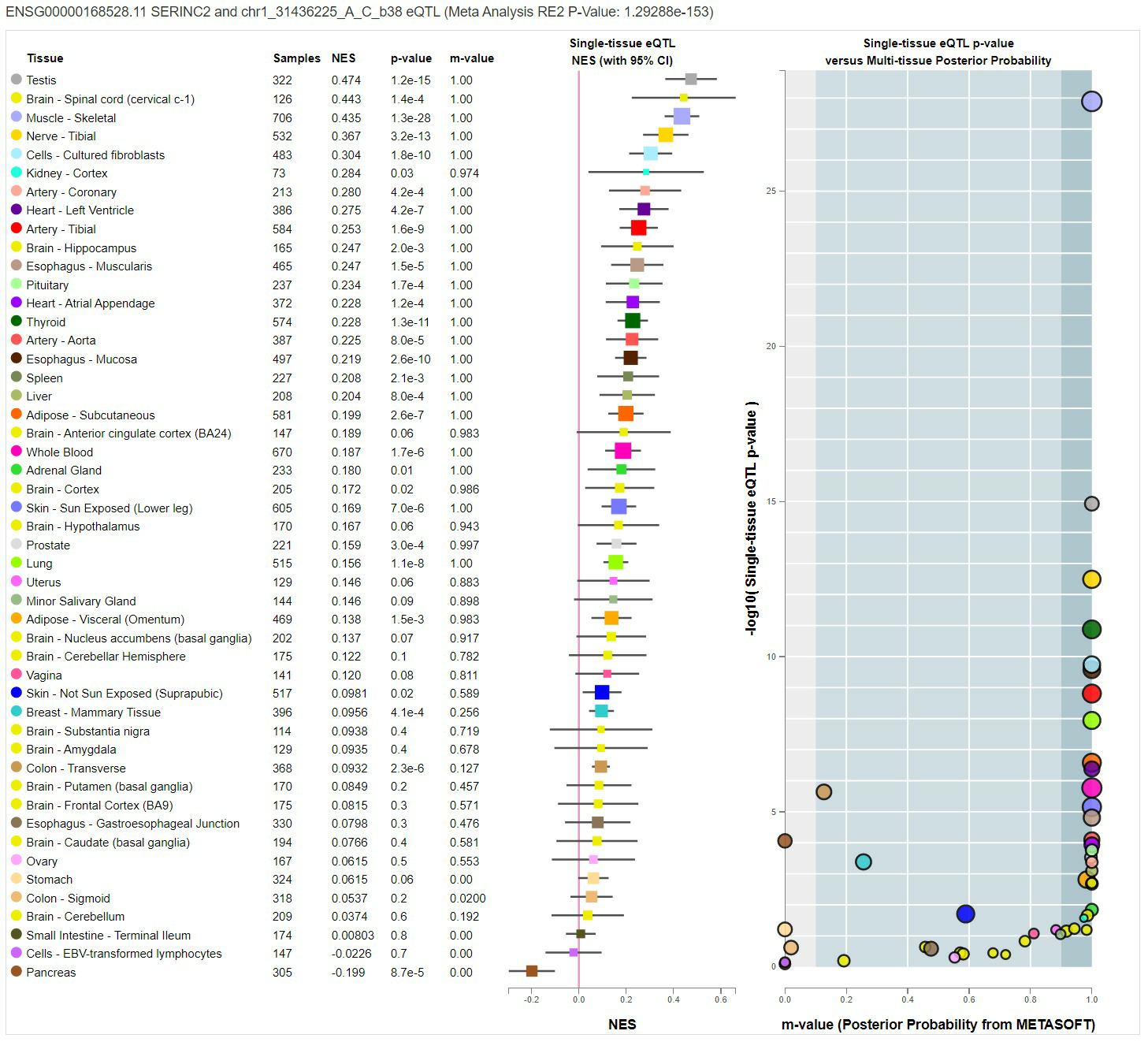

Supplement: S2 Fig — NES is the normalized effect size (β) from single-tissue eQTL analysis. P-value is from the t-test that compares observed NES in single-tissue eQTL analysis to the null hypothesis of no NES. m-value represents a posterior probability that the effect of eQTL exists in each tissue of a cross-tissue meta-analysis. (TIF) [file pone.0278392.s005.tif]

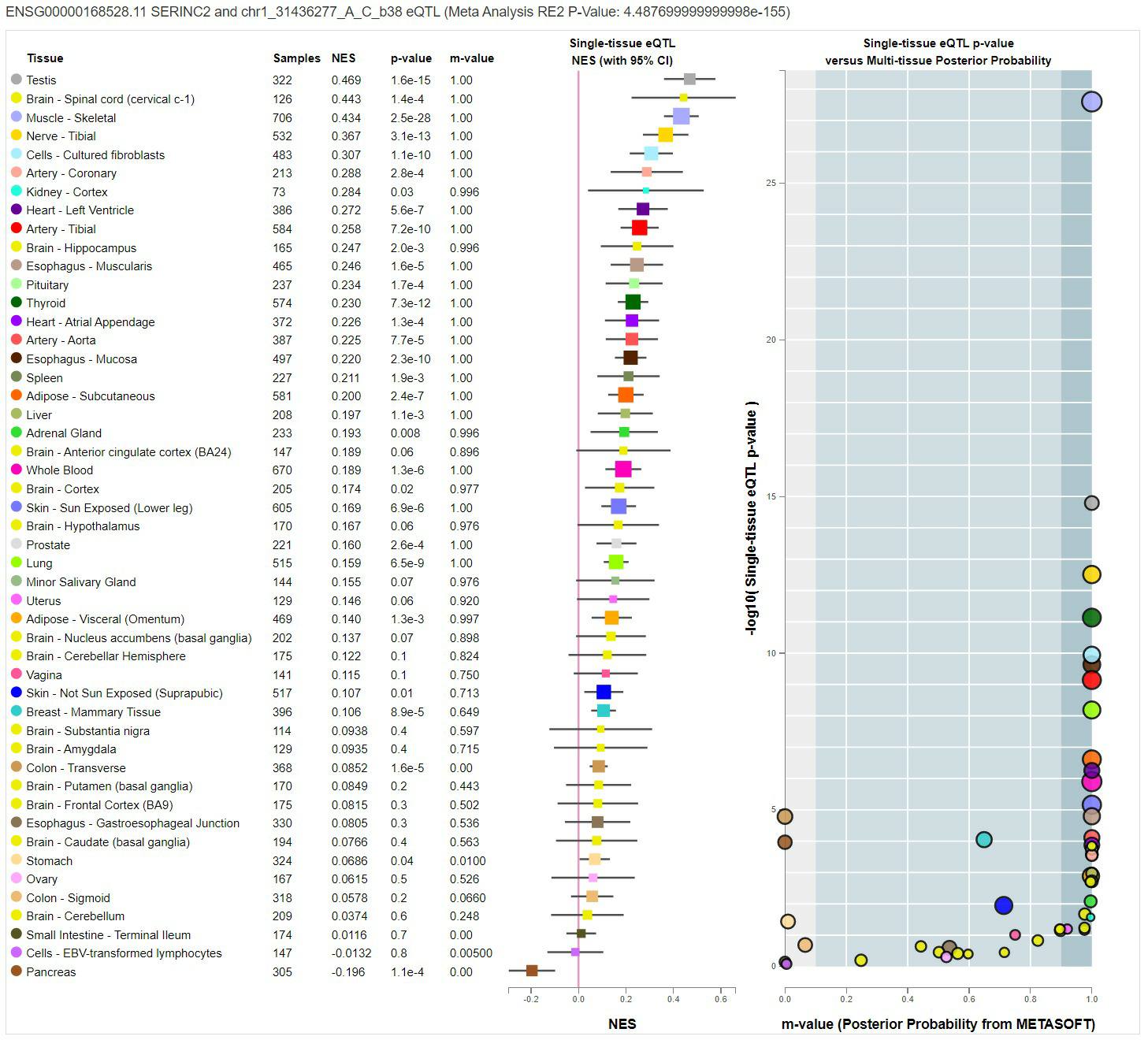

Supplement: S3 Fig — NES is the normalized effect size (β) from single-tissue eQTL analysis. P-value is from the t-test that compares observed NES in single-tissue eQTL analysis to the null hypothesis of no NES. m-value represents a posterior probability that the effect of eQTL exists in each tissue of a cross-tissue meta-analysis. (TIF) [file pone.0278392.s006.tif]
